# Effects of enriched endogenous omega-3 fatty acids on age-related hearing loss in mice

**DOI:** 10.1186/s13104-019-4809-8

**Published:** 2019-11-26

**Authors:** Yohei Honkura, Jun Suzuki, Nobuyuki Sakayori, Hitoshi Inada, Tetsuaki Kawase, Yukio Katori, Noriko Osumi

**Affiliations:** 1 Department of Otolaryngology-Head and Neck Surgery, Tohoku University School of Medicine, 1-1 Seiryo-machi, Aoba-ku, Sendai, Miyagi 980-8574 Japan; 2 Department of Molecular Genetics, Institute of Biomedical Sciences, Fukushima Medical University, 1 Hikarigaoka, Fukushima, 960-1295 Japan; 3 Department of Developmental Neuroscience, Centers for Neuroscience, Tohoku University School of Medicine, 2-1 Seiryo-machi, Aoba-ku, Sendai, Miyagi 980-8574 Japan; 4 Laboratory of Rehabilitative Auditory Science, Tohoku University Graduate School of Biomedical Engineering, 1-1 Seiryou-machi, Aoba-ku, Sendai, Miyagi 980-8574 Japan

**Keywords:** Omega-3 (n-3) fatty acids, Cochlea, Age-related hearing loss, C57BL/6 mouse

## Abstract

**Objective:**

Dietary intervention is a practical prevention strategy for age-related hearing loss (AHL). Omega-3 (n-3) polyunsaturated fatty acids (PUFAs) may be effective in prevention of AHL due to their anti-inflammatory and tissue-protective functions. Age-related changes in the hearing function of wild-type and *Fat*-*1* transgenic mice derived from the C57BL/6N strain, which can convert omega-6 PUFAs to n-3 PUFAs and consequently produce enriched endogenous n-3 PUFAs, were investigated to test the efficacy of n-3 PUFAs for AHL prevention.

**Results:**

At 2 months, the baseline auditory brainstem response (ABR) thresholds were the same in *Fat*-*1* and wild-type mice at 8–16 kHz but were significantly higher in *Fat*-*1* mice at 4 and 32 kHz. In contrast, the ABR thresholds of *Fat*-*1* mice were significantly lower at 10 months. Moreover, the ABR thresholds of *Fat*-*1* mice at low-middle frequencies were significantly lower at 13 months (12 kHz). Body weights were significantly reduced in *Fat*-*1* mice at 13 months, but not at 2, 10, and 16–17 months. In conclusion, enriched endogenous n-3 PUFAs produced due to the expression of the *Fat*-*1* transgene partially alleviated AHL in male C57BL/6N mice.

## Introduction

Age-related hearing loss (AHL) is the most common cause of sensorineural hearing loss in adults and is one of the most prevalent age-related physical conditions [1, 2]. There are currently no established preventions or treatments for AHL, even though it is a high priority issue. Nutritional improvement is one potential intervention, and omega-3 (n-3) polyunsaturated fatty acids (PUFAs), such as docosahexaenoic acid (DHA), are promising candidates for AHL prevention due to their potential prevention of cognitive decline [3]. In addition, anti-inflammatory and pro-resolving metabolites of n-3 PUFAs are known to have protective effects in neurological disorders [4].

Several studies have reported positive effects of n-3 PUFAs on hearing function in humans, [5–7] while perinatal diets supplemented with high levels of DHA or n-3 PUFAs have negative effects on the auditory systems of rat pups [8, 9] and adult rat offspring [10]. Therefore, the effects of n-3 PUFAs on hearing function remain unclear, particularly due to the unavoidable genetic and environmental non-uniformity of human subjects.

In this study, we investigated the preventive effects of enriched endogenous n-3 PUFAs on the progression of AHL in *Fat*-*1* transgenic mice (*Fat*-*1* mice). The *Fat*-*1* mice express the nematode-derived *Fat*-*1* gene encoding an enzyme to convert omega-6 (n-6) to n-3 PUFAs [11]. Thus, investigation of *Fat*-*1* mice provides more reliable and more definitive results than the studies using conventional dietary supplementation of PUFAs.

## Main text

### Methods

#### Animals and genotyping

Wild-type (WT) C57BL/6N mice were purchased from CLEA Japan. Heterozygous *Fat*-*1* mice [11] were mated with WT mice, and their male offspring were used. Mice were maintained on a normal diet (CE-2, CLEA Japan, Tokyo, Japan) with water ad libitum and housed under a standard 12 h light/12 h dark schedule.

Genotyping was performed as previously described [12]. The following primers were used to amplify the *Fat*-*1* transgene: forward 5′-CACCAACCACATCGACAAAG-3′ and reverse 5′-CGACGTGCTGCAGATAGGTA-3′. Polymerase chain reaction amplification was performed for 30 cycles under the following parameters: denaturation at 95 °C for 30 s, annealing at 55 °C for 30 s, and extension at 72 °C for 2 min.

#### Cochlear function testing

Auditory brainstem responses (ABR) were recorded as previously described [13]. The animals were anesthetized with intraperitoneal injection of ketamine (100 mg/kg) and xylazine (20 mg/kg). Needle electrodes were placed subcutaneously at the vertex, the base of the pinna, and the back. ABR recordings were obtained using a TDT System 3 hardware and BioSigRP software (Tucker-Davis Technologies, Alachua, FL). ABRs were evoked with tone bursts of pure tones at frequencies of 4, 8, 12, 16, and 32 kHz, which were generated using SigGenRP software and a digital-to-analog converter (RP2.1). ABRs were relayed to a programmable attenuator (PA5), an amplifier (SA1), and a closed-field loudspeaker (CF1). The electrode outputs were delivered to an alternating current preamplifier (P55, Astro-Med, West Warwick, RI) and amplified (× 100). Evoked responses were filtered with a band pass of 10–3000 Hz and were averaged with 1000 sweeps. Responses were collected for stimulus levels in 5-dB steps from sound pressure levels (SPLs) of 100 dB to 10 dB. ABR threshold was defined as the lowest sound level at which reproducible waveforms could be observed. If no response was obtained at 100 dB SPL, the ABR threshold was defined as 105 dB SPL.

#### Tissue preparation

The mice were anesthetized with an intraperitoneal injection of ketamine (100 mg/kg) and xylazine (20 mg/kg). The anesthetized mice were then transcardially perfused with 4% paraformaldehyde (P6148, Sigma-Aldrich, St. Louis, MO) in phosphate-buffered saline (PBS). The cochleae were removed and post-fixed in 4% paraformaldehyde overnight at 4 °C. The fixed cochleae were decalcified in 10% ethylenediaminetetra-acetic acid disodium salt dehydrate (345-01865, Dojindo, Mashiki, Japan) for 2 days at 4 °C.

#### Cochlear whole-mount

Microdissected cochlear pieces were blocked in 5% normal horse serum in PBS and 0.3% Triton X-100 for 10 min at room temperature and stained with a rhodamine–phalloidin probe (1:250, Cytoskeleton, Denver, CO) at room temperature for 30 min. Cochlear pieces were slide-mounted using Vectashield (Vector Labs, Burlingame, CA). Cochlear pieces were imaged using a fluorescence microscope (E800, Nikon, Tokyo, Japan). Stained cochlear whole-mounts were imaged using confocal microscopy (TCS SP5, Leica, Wetzlar, Germany). Quantitative results were obtained by evaluating 90 outer hair cells (OHCs) across three rows in a given microscopic field.

#### Histological analysis of spiral ganglion (SG) neurons and stria vascularis (SV)

Decalcified tissues were embedded in paraffin, and coronal Sects. (3 µm) were cut and mounted. The sections were stained with hematoxylin and eosin and visualized using a light microscope (BZ-9000, Keyence, Osaka, Japan). Three cochlear regions were used for evaluation of cochlear histology. Three sections per animal were used to calculate mean numbers. Area measurements and cell counts were performed using BZ-H1C (Keyence). The SG area was calculated for each section, and the SG neurons were counted. Three measurements of SV thickness were obtained from each image and were averaged.

#### Statistical analysis

Statistical analyses were conducted using StatMate IV (ATMS Company, Tokyo, Japan) or JMP^®^ 12 (SAS Institute Inc., Cary, NC). All data are presented as means ± standard errors of the mean. The two-sample *t*-tests and two-way analyses of variance (ANOVA) followed by Bonferroni post hoc tests were used. *P* values < 0.05 after the Bonferroni correction were considered statistically significant.

### Results

#### Time course of ABR thresholds and body weights

**Fig. 1:**
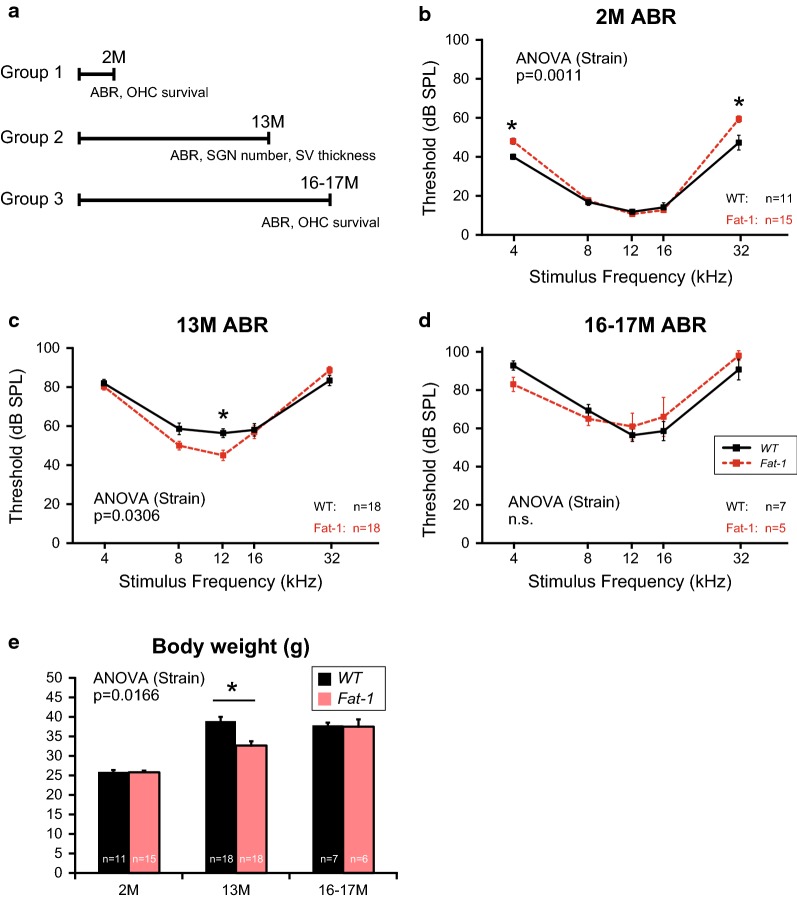
Chronological changes in auditory brainstem response (ABR) thresholds and body weights. **a** Schematic timeline of the experimental protocol. **b**–**d** Mean ABR thresholds (± standard errors of the mean) for each time point (**b** 2 months, **c** 13 months, and **d** 16–17 months). Animal numbers were as follows: Wild-type (WT), n = 11; *Fat*-*1*, n = 15 in **b** WT, n = 18; *Fat*-*1*, n = 18 in **c**; and WT, n = 7; *Fat*-*1*, n = 5 in **d**. In the early stage (2 months), ABR thresholds were significantly elevated in *Fat*-*1* mice compared with WT mice in response to stimuli at 4 and 32 kHz (**b**). In contrast, ABR thresholds of *Fat*-*1* mice were significantly decreased at low-middle frequencies at 13 months (**c**). Time course of changes in body weight at 2 months (wild-type [WT], n = 11; *Fat*-*1*, n = 15), 13 months (WT, n = 18; *Fat*-*1*, n = 18), and 16–17 months (WT, n = 7; *Fat*-*1*, n = 6) of age. **e** Body weights of WT and *Fat*-*1* mice were not different in the early stage, but those of *Fat*-*1* mice were significantly decreased at 13 months. No significant difference was observed between the two groups in the later stage (at 16–17 months). *P* values < 0.05 were considered statistically significant (*).* OHC* outer hair cell,* SGN* spiral ganglion neuron,* SPL*  sound pressure level,* SV* stria vascularis,* ANOVA*  analysis of variance,* ns* no significant difference

The C57BL/6N mice exhibit early onset and progression of AHL mainly due to a single nucleotide variant in the *Cdh23* gene (*Cdh23*^753A^) [14, 15]. *Fat*-*1* mice have a *Cdh23*^753A/753A^ genotype for *Cdh23* (Additional file 1: Fig S1). To determine the effects of enriched endogenous n-3 PUFAs on AHL progression, ABR thresholds were first measured at 2, 13, and 16–17 months of age (Fig. 1a). Although there were no differences in the ABR thresholds in response to 8-, 12-, or 16-kHz stimuli, the ABR thresholds were significantly higher in response to 4-kHz and 32-kHz stimuli in *Fat*-*1* mice at 2 months (Fig. 1b). Unlike at 2 months, the ABR thresholds at 13 months were significantly lower at 12 kHz in *Fat*-*1* mice (Fig. 1c). No significant differences were observed at 16–17 months (Fig. 1d).

WT and *Fat*-*1* mice in the same colony were weighed at 2, 13, and 16–17 months to evaluate the effect of enriched endogenous n-3 PUFAs on body weight, Although the body weights of WT mice increased and reached a plateau at 13 months, the body weights of *Fat*-*1* mice increased more slowly and reached the same level as that reached by WT mice at 16–17 months (Fig. 1e). The body weights of *Fat*-*1* mice were significantly lower than those of WT mice at 13 months (Fig. 1e). Therefore, enriched endogenous n-3 PUFAs slowed age-related weight gain.

**Fig. 2:**
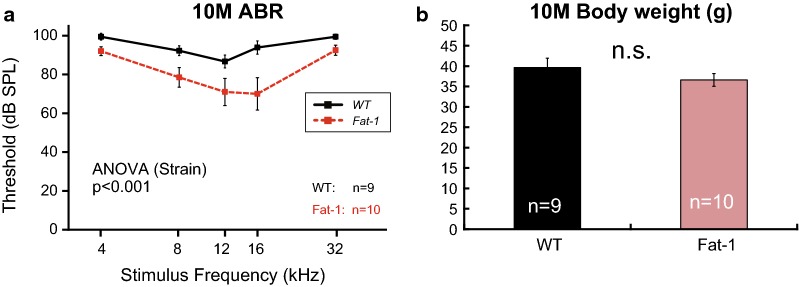
Auditory brainstem response (ABR) thresholds and body weights at 10 months of age. **a** Mean ABR thresholds (± standard errors of the mean) at 10 months. ABR thresholds for both wild-type (WT) and *Fat*-*1* transgenic mice (*Fat*-*1*) were increased when compared to those of other groups (13 and 16–17 months) due to unknown reasons. The animal numbers were as follows: WT, n = 9 and *Fat*-*1*, n = 10. Although there were no significant differences in stimulus frequency as indicated in the Bonferroni post hoc test, the two-way analysis of variance (ANOVA) revealed that the main effect (strain) was statistically significant. **b** Body weights at 10 months (WT, n = 9; *Fat*-*1*, n = 10). *P* values < 0.05 were considered to statistically significant (*).* ns* no significant difference

We additionally evaluated the ABR thresholds at 10 months using a different colony. Although ABR thresholds for both WT and *Fat*-*1* mice were increased compared to those of other groups (13 and 16–17 months) due to unknown reasons, we found that the ABR thresholds were significantly different between the two strains at 10 months (Fig. 2a, Additional file 1: Tables S1). These results suggest that enriched endogenous n-3 PUFAs delayed the early progression of AHL.

#### OHC survival rates at 2 and 16–17 months

**Fig. 3:**
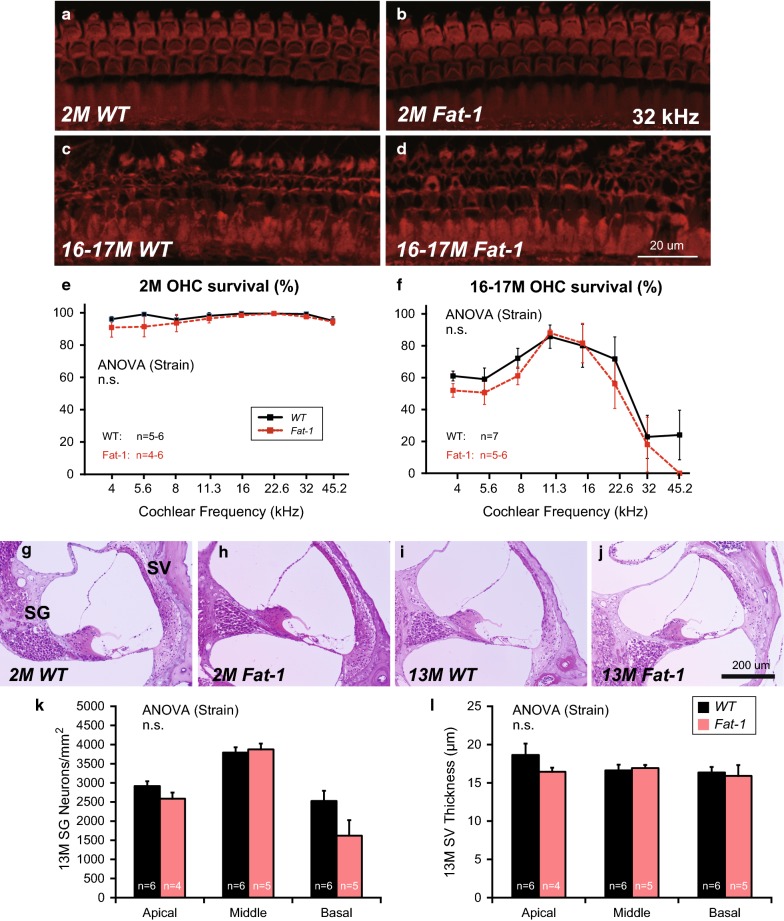
Cochlear histology. **a**–**d** Representative images of organs of Corti in the 32-kHz region [**a** wild-type (WT) mice at 2 months, **b** *Fat*-*1* mice at 2 months, **c** WT mice at 16–17 months, and **d** *Fat*-*1* mice at 16–17 months]. At 32 kHz, almost all OHCs were intact in the early stage (**a**, **b**), but were severely damaged at 16–17 months in both WT and *Fat*-*1* mice (**c**, **d**). **e** Quantitative data for OHC survival at 2 months. **f** Quantitative data of OHC survival at 16–17 months. **g**–**j** Representative images of the cochlear middle turn (**g** wild-type [WT] mice at 2 months, **h** *Fat*-*1* mice at 2 months, **i** WT mice at 16–17 months, and **j** *Fat*-*1* mice at 16–17 months). There were no apparent differences between these two groups at either 2 or 13 months. **k** Quantitative data for spiral ganglion (SG) neuron counts at 13 months. **l** Quantitative data for stria vascularis (SV) thickness measurements at 13 months. *P* values < 0.05 were considered statistically significant (*).* ns* no significant difference

Representative cochlear whole-mount images from WT and *Fat*-*1* mice in the 32-kHz region at 2 and 16–17 months are shown in Fig. 3a–d. Almost all OHCs were intact in both WT and *Fat*-*1* mice at 2 months (Fig. 3a, b). In contrast, OHCs were severely damaged in both WT and *Fat*-*1* mice at 16–17 months (Fig. 3c, d). OHC survival rates were not significantly different between these two groups at 2 months and 16–17 months (Fig. 3e, f). In summary, OHC survival did not significantly differ between the two groups at young (2 months) and old (16–17 months) stages.

#### Histological changes in cochleae at 2 and 13 months

Cochlear coronal sections were histologically investigated at 2 and 13 months to assess the effects of enriched endogenous n-3 PUFAs on cochlear degeneration. No apparent cochlear degradations were observed in the SG, SV, or spiral ligament of WT or *Fat*-*1* mice at 2 months (Fig. 3g, h). Although several indicators of cochlear degeneration were observed, no apparent differences were found between WT or *Fat*-*1* mice at 13 months (Fig. 3i, j). The number of SG neurons and the thickness of the SV showed no significant differences between the two groups at 13 months (Fig. 3k, l). Therefore, no apparent differences in age-related cochlear degeneration were found between WT and *Fat*-*1* mice at 13 months.

### Discussion

Dietary intervention may be a promising prophylaxis for AHL, as diet is one modifiable risk factors for AHL [16]. Here we used *Fat*-*1* mice to evaluate the effects of n-3 PUFAs without the many problems associated with dietary supplementation such as oxidation of PUFAs in food pellets. We found that enriched endogenous n-3 PUFAs suppressed age-related body weight gain and partially slowed the progression of AHL in male C57BL/6N mice. Recently, long-term dietary n-3 PUFA supplementation was shown to ameliorate the progression of AHL in female C57BL/6J mice [17]. In that study, ABR thresholds in response to 4-, 8-, and 40-kHz stimuli were significantly lower in mice administered n-3 PUFA at 10 months [17]. This protective effect during the early aging period seems to be reproducible, as we also observed a similar protective effect in male *Fat*-*1* mice at 10 months (Fig. 2a). Together, these observations may suggest that n-3 PUFAs are effective for AHL prevention in C57BL/6 mice during the early aging period.

In this study, the progression of AHL was significantly slowed in *Fat*-*1* mice only in response to low-middle frequencies. This may have been due to baseline differences in ABR threshold at 2 months. Considering that the ABR thresholds of *Fat*-*1* mice at 10 months tended to be lower than those in WT mice (Fig. 2a), enriched endogenous n-3 PUFAs may have protective effects on a wider range of cochlear frequency regions. However, it is also possible that sensitivity to n-3 PUFAs varies in different cochlear regions. We have already observed such a regional difference: maternal consumption of a diet high in n-6 and deficient in n-3 has been reported to severely impair neocortical development in the rostral region, although the effect is mild in the caudal region [12]. n-3 PUFA receptors and transporters may have a gradient expression in the cochlea. Further investigation is required to increase our knowledge of PUFA-mediated molecular mechanisms in the cochlea, and *Fat*-*1* mice will be useful for future studies.

In addition to the positive effects of enriched endogenous n-3 PUFAs for AHL prevention, we also noticed negative effects on hearing development (Fig. 1b). These negative effects of n-3 PUFAs are consistent with previous studies using rats: maternal dietary supplementation of DHA had negative effects on auditory brainstem conduction times in pups [8, 9]. In addition, excess maternal intake of n-3 PUFAs was shown to cause abnormal ABRs in older adult offspring [10, 18]. Further structural and functional analyses will be useful to reveal the mechanisms underlying the potential adverse effects of n-3 PUFAs.

## Limitations

This study is not without limitations. First, only male mice were used in this study, so it is not known how the present findings would apply to females. Second, ABR thresholds of both WT and *Fat*-*1* mice at 10 months were increased due to unknown reasons. Considering the fact that we performed the ABR measurements on 10-month-old mice more than 2 years after the initial ABR experiments (2, 13, 16–17 months old), we believe that the higher ABR thresholds at 10 months were likely caused by variability in epigenetic backgrounds related to the progression of AHL within the same C57BL/6 strain. Third, this study lacks the data of metabolome analysis because of technical problems. In this study, WT and *Fat*-*1* mice were fed a standard rodent diet (CE2, Additional file 1: Table S2). As a result, the cochlear n-6/n-3 ratio was predicted to be higher in WT mice than in *Fat*-*1* mice. Since the n-6/n-3 ratio of *Fat*-*1* mice is close to 1:1 in all organs/tissues examined [11], we believe that the ratio is also close to 1:1 in *Fat*-*1* mouse cochlea. Future studies should establish metabolomic data using cochlear tissue. Finally, a limitation of our histological analysis was the smaller animal numbers and larger variations in histological analyses that occurred compared with ABR analyses. Regarding the lack of correlation between the ABR and histological results, we suspect that the n-3 PUFAs help to protect a wide range of the cochlea, not just on a specific cochlear region. However, it is also possible that additional histological assessments might reveal other reasons for the otoprotective effect of the n-3 PUFAs. Unfortunately, we are unable to conduct further animal experiments currently. The mechanism of the otoprotective effects of the n-3 PUFAs should be investigated further in future studies.

## Supplementary information

**Additional file 1: Fig. S1.** *Cdh23* genotyping and sequencing of *Fat*-*1* mice. The *Cdh23* gene in three *Fat*-*1* transgenic mice was sequenced. All of the *Fat*-*1* transgenic mice examined had the same *Cdh23*^753A/753A^ genotype. **Table S1.** Summary table for the two-way ANOVA. **Table S2**. Fatty acid composition of CE-2 diet. **Additional methods.** *Cdh23* genotyping.

## Data Availability

Datasets used and analyzed for the current study are available from the corresponding author upon reasonable request.

## Abbreviations

ABR: auditory brainstem response
AHL: age-related hearing loss
ANOVA: analyses of variance
DHA: docosahexaenoic acid
n.s.: no significant difference
OHC: outer hair cell
PUFA: polyunsaturated fatty acid
SG: spiral ganglion
SGN: spiral ganglion neuron
SPL: sound pressure level
SV: stria vascularis
WT: wild-type
