# Supplementary material for: Effects of enriched endogenous omega-3 fatty acids on age-related hearing loss in mice
Source: BMC Res Notes. 2019 Nov 26;12:768. doi: 10.1186/s13104-019-4809-8 (PMC6878677; doi:10.1186/s13104-019-4809-8)
Supplement: Supplementary file 1 — Additional file 1: Fig. S1. Cdh23 genotyping and sequencing of Fat-1 mice. The Cdh23 gene in three Fat-1 transgenic mice was sequenced. All of the Fat-1 transgenic mice examined had the same Cdh23753A/753A genotype. Table S1. Summary table for the two-way ANOVA. Table S2. Fatty acid composition of CE-2 diet. Additional methods. Cdh23 genotyping. [file 13104_2019_4809_MOESM1_ESM.pdf]

Fig. S1. *Cdh23* genotyping and sequencing of *Fat-1* mice.

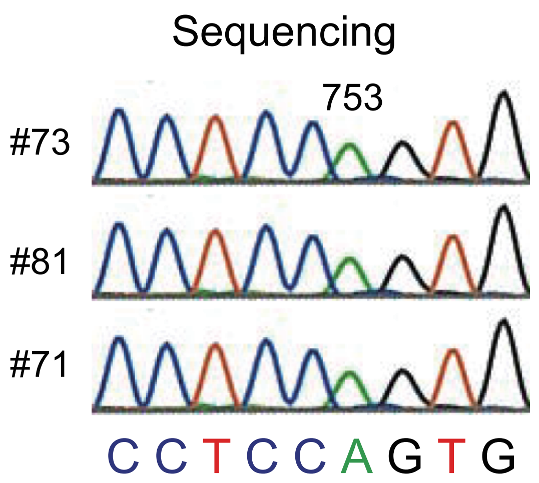

Table S1. Summary table for the two-way ANOVA.

Fig. 1B

Source of Variation	Degrees of Freedom	Sum of Squares	F Ratio	P Value
Strain	1	426.603	11.2518	0.0011
Stimulus Frequency	4	38240.047	252.149	<0.0001
Strain * Stimulus Frequency	4	928.508	6.1224	0.0002

Fig. 1C

Source of Variation	Degrees of Freedom	Sum of Squares	F Ratio	P Value
Strain	1	551.25	4.7533	0.0306
Stimulus Frequency	4	38536.944	83.0737	<0.0001
Strain * Stimulus Frequency	4	1570.278	3.385	0.0108

Fig. 1D

Source of Variation	Degrees of Freedom	Sum of Squares	F Ratio	P Value
Strain	1	17.09	0.1332	0.7165
Stimulus Frequency	4	13262.69	25.8408	<0.0001
Strain * Stimulus Frequency	4	773.459	1.507	0.2128

Fig. 1E

Source of Variation	Degrees of Freedom	Sum of Squares	F Ratio	P Value
Strain	1	78.6594	6.0265	0.0166
Age	2	1856.9686	71.1355	<0.0001
Strain * Age	2	171.9292	6.5862	0.0024

Fig. 2A

Source of Variation	Degrees of Freedom	Sum of Squares	F Ratio	P Value
Strain	1	4532.99	24.7196	<0.0001
Stimulus Frequency	4	4948.2461	6.746	<0.0001
Strain * Stimulus Frequency	4	951.9461	1.2978	0.2769

Table S1. Summary table for the two-way ANOVA.

Fig. 3E

Source of Variation	Degrees of Freedom	Sum of Squares	F Ratio	P Value
Strain	1	84.62137	0.0481	0.8267
Cochlear Frequency	7	739.12335	0.0601	0.9997
Strain * Cochlear Frequency	7	89.19892	0.0072	1

Fig. 3F

Source of Variation	Degrees of Freedom	Sum of Squares	F Ratio	P Value
Strain	1	2877.697	3.179	0.0769
Cochlear Frequency	7	55730.092	8.795	<0.0001
Strain * Cochlear Frequency	7	2025.967	0.3197	0.944

Fig. 3K

Source of Variation	Degrees of Freedom	Sum of Squares	F Ratio	P Value
Strain	1	116.046	4.1698	0.0514
Area	2	1718.6094	30.8766	<0.0001
Strain * Area	2	134.3344	2.4135	0.1093

Fig. 3L

Source of Variation	Degrees of Freedom	Sum of Squares	F Ratio	P Value
Strain	1	4.764406	1.0556	0.3137
Area	2	10.222521	1.1325	0.3376
Strain * Area	2	8.354084	0.9255	0.409

Table S2. Fatty Acid Composition of CE-2 Diet.

	CE-2 (%)
C12:0	n.d.
C14:0	0.82
C14:1	n.d.
C15:0	n.d.
C16:0	16.25
C16:1n-7	1.34
C17:0	0.52
C17:1	n.d.
C18:0	2.13
C18:1n-9 cis	20.95
C18:1n-9 trans	n.d.
C18:1n-7	2.11
C18:2n-6 cis	44.44
C18:2n-6 trans	n.d.
C18:3n-6	n.d.
C18:3n-3	3.26
C20:0	0.49
C20:1n-9	0.72
C20:4n-6	n.d.
C20:5n-3	2.27
C22:0	0.25
C22:5n-3	n.d.
C22:6n-3	1.29
C24:0	0.18
C24:1	0.2
Unidentified	2.78
Total	100
Total n-6	44.44
Total n-3	6.82
n-6/n-3 ratio	6.52

n.d.=not detected.

Reprinted from Experimental Animal Diet Data Collection (2011 edition) with permission from Clea Japan.

Additional methods

Cdh23 genotyping

To confirm that the *Fat-1* transgenic mice had the *Cdh23*$^{753A/753A}$ genotype for *Cdh23*, which is common in the C57BL6 strain and accelerates age-related hearing loss, we isolated genomic DNA from three *Fat-1* transgenic mice, amplified the DNA fragments by PCR, and then sequenced the region of DNA containing the 753rd nucleotide in the *Cdh23* gene . The primer sequences were as follows:

Forward: 5'- GATCAAGACAAGACCAGACCTCTGTC-3'

Reverse: 5'-GAGCTACCAGGAACAGCTTGGGCCTG-3'

The cycling conditions for PCR were as follows: 95°C for 2 min; 35 cycles of 95°C for 10 sec, 63.7°C for 10 sec, 72°C for 30 sec; and 72°C for 5 min.

The expected band size of the PCR product was 360 bp. The *Cdh23* gene was sequences in three *Fat-1* transgenic mice for both strands.
